# A triclinic polymorph of bis­(μ_2_-ethane­thiol­ato)-1:2κ^2^ *S*:*S*;3:4κ^2^ *S*:*S*-(μ_4_-disulfido-1:2:3:4κ^4^ *S*:*S*:*S*′:*S*′)tetra­kis­[tricarbonyl­iron(II)](2 *Fe*—*Fe*)

**DOI:** 10.1107/S1600536811017405

**Published:** 2011-05-14

**Authors:** Youtao Si, Hui Chen, Chang Neng Chen

**Affiliations:** a Key Laboratory of Humid Subtropical Eco-Geographical Processes, Ministry of Education, Fujian Normal University, Fuzhou 350007, People’s Republic of China; b State Key Laboratory of Structural Chemistry, Fujian Institute of Research on the Structure of Matter, Fuzhou, Fujian 350002, People’s Republic of China

## Abstract

Next to the monoclinic polymorph [Cheng *et al.* (2005 ▶). *Acta Cryst.* E**61**, m892–m894], the triclinic title compound, [Fe_4_(C_2_H_5_S)_2_(S_2_)(CO)_12_], is the second known form of this composition. The structure is composed of an [Fe_2_(C_2_H_5_S)(S)(CO)_6_] subcluster, which is linked to its counterpart by an inversion centre located at the mid-point of the central disulfide bond. The Fe_2_S_2_ core of each subcluster exhibits a butterfly-like shape, with two S atoms bridging two Fe atoms. In the subcluster, each Fe atom is coordinated in a distorted octa­hedral coordination by three terminal carbonyl C atoms, two S atoms and one Fe atom. The crystal packing is accomplished through van der Waals inter­actions.

## Related literature

For more details about hydrogenases, including Fe—Fe hydrogenases, see: Darensbourg *et al.* (2000 ▶). Two procedures are mainly used for the synthesis of model compounds containing the Fe_2_S_2_ subcluster of Fe—Fe hydrogenases, see: Lawrence *et al.* (2001 ▶); Li & Rauchfuss (2002 ▶). The monoclinic polymorph (space group *P*2_1_/*c*) of the title compound has been reported by Cheng *et al.* (2005 ▶).
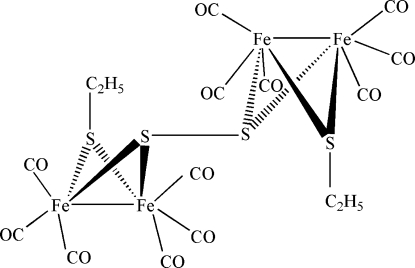


## Experimental

### 

#### Crystal data

[Fe_4_(C_2_H_5_S)_2_(S_2_)(CO)_12_]
*M* *_r_* = 745.88
Triclinic, 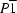

*a* = 8.365 (4) Å
*b* = 9.296 (5) Å
*c* = 10.209 (5) Å
α = 87.57 (2)°
β = 70.082 (17)°
γ = 66.103 (17)°
*V* = 678.2 (6) Å^3^
*Z* = 1
Mo *K*α radiation
μ = 2.46 mm^−1^
*T* = 293 K
0.15 × 0.12 × 0.03 mm

#### Data collection

Rigaku Mercury70 CCD diffractometer
Absorption correction: multi-scan (*CrystalClear*; Rigaku, 2002 ▶) *T*_min_ = 0.771, *T*_max_ = 1.000
5323 measured reflections
3060 independent reflections
1939 reflections with *I* > 2σ(*I*)
*R*_int_ = 0.036

#### Refinement

*R*[*F* ^2^ > 2σ(*F* ^2^)] = 0.048
*wR*(*F* ^2^) = 0.100
*S* = 0.99
3060 reflections
163 parameters
H-atom parameters constrained
Δρ_max_ = 0.39 e Å^−3^
Δρ_min_ = −0.42 e Å^−3^

### 

Data collection: *CrystalClear* (Rigaku, 2002 ▶); cell refinement: *CrystalClear*; data reduction: *CrystalClear*; program(s) used to solve structure: *SHELXS97* (Sheldrick, 2008 ▶); program(s) used to refine structure: *SHELXL97* (Sheldrick, 2008 ▶); molecular graphics: *WinGX* (Farrugia, 1999 ▶); software used to prepare material for publication: *publCIF* (Westrip, 2010 ▶).

## Supplementary Material

Crystal structure: contains datablocks I, global. DOI: 10.1107/S1600536811017405/wm2485sup1.cif

Structure factors: contains datablocks I. DOI: 10.1107/S1600536811017405/wm2485Isup2.hkl

Additional supplementary materials:  crystallographic information; 3D view; checkCIF report

## Figures and Tables

**Table 1:** Selected bond lengths (Å)

Fe1—C1	1.786 (5)
Fe1—C2	1.793 (5)
Fe1—C3	1.824 (5)
Fe1—S1	2.2393 (15)
Fe1—S2	2.2688 (16)
Fe1—Fe2	2.5183 (15)
Fe2—C5	1.789 (5)
Fe2—C6	1.794 (5)
Fe2—C4	1.806 (5)
Fe2—S1	2.2457 (15)
Fe2—S2	2.2711 (18)
S1—S1^i^	2.113 (2)

Symmetry code: (i) 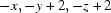.

**Fig. 1.**
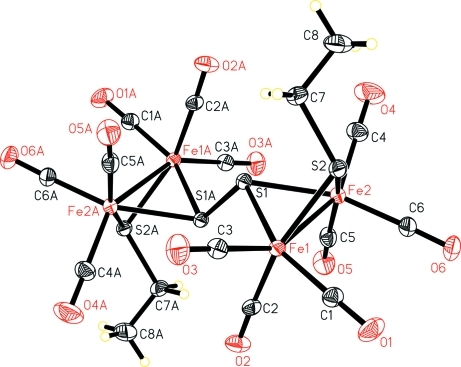
The molecular structure of (I), with atom labels and 20% probability displacement ellipsoids for all non-H atoms. [Symmetry operator A: -x, -y + 2, -z + 2.]

**Fig. 2.**
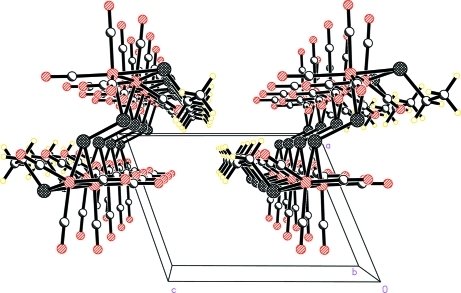
The packing diagram of (I), viewed down the b axis.

## supplementary crystallographic information

### Comment

Fe—Fe hydrogenases are enzymes capable of efficiently catalysing the reversible transformation between H^+^ and H_2_ (Darensbourg *et al.*, 2000). Chemists have been trying to achieve H_2_ production technologies of practical use by studying the catalytic process by such kind of hydrogenases, aiming at solving the current energy problem. The well known active site of Fe—Fe hydrogenases, established by X-ray crystallographic and spectroscopic techniques, has an Fe_2_S_2_ cluster linked to a Fe_4_S_4_ cuboidal unit by a cysteine-S atom. While the Fe_4_S_4_ unit is assumed to be reponsible for transferring eletrons, the Fe_2_S_2_ cluster plays an important role in the catalysis process. Thus, many works concentrate on compounds containing the Fe_2_S_2_ cluster.

Two kinds of procedures are frequently used to synthesize model substances of the Fe_2_S_2_ cluster, *e.g.* Fe_2_(SCH_2_)_2_N*R*(CO)_6_. The first procedure is a condensation of (ClCH_2_)_2_N*R* and Li_2_[Fe_2_S_2_(CO)_6_], and the second is a condensation of Fe_2_(SH)_2_(CO)_6_ with formaldehyde in the presence of primary amines (Lawrence *et al.*, 2001; Li & Rauchfuss, 2002). In both cases, LiEt_3_BH are used to cleave the S—S bond of the starting material Fe_2_S_2_(CO)_6_. When trying to get some new complexes using the first procedure, we found some by-products which reflect the diversity of the reactivity of (FeS)_n_ clusters. Here we report a triclinic polymorph, (I), of [Fe_4_(C_2_H_5_S)_2_(S_2_)(CO)_12_]. Another monoclinic polymorph (space group *P*2_1_/*c*) has been reported previously (Cheng *et al.*, 2005).

As can be seen in Fig. 1, the crystallographically imposed center of inversion is located at the mid-point of the S1—S1A bond, and thus the asymmetric unit contains one half of the [Fe_4_(C_2_H_5_S)_2_(S_2_)(CO)_12_] formula unit. The two Fe atoms of the asymmetric unit (Fe1, Fe2) are linked through an Fe—Fe single bond and are bridged by two S atoms (S1, S2). Thus a butterfly-like arrangement is formed, with a dihedral angle between the two Fe_2_S planes being 100.53 (6)°. The average Fe—S bond length is 2.256 (16) Å, and the average Fe—S—Fe angle is 67.9 (6)°. The octahedral coordination geometry around each Fe atom is completed by three carbonyl C atoms [average Fe—C distance 1.799 (14) Å, average C—Fe—C angle 97 (4)°].

The packing diagram is shown in Fig. 2. There is only one molecule in each unit cell, and neighbouring molecules pack along the *a* axis; the crystal is stabilized by van der Waals interactions.

In comparison with the monoclinic polymorph (Cheng *et al.*, 2005), the configuration of the [Fe_4_(C_2_H_5_S)_2_(S_2_)(CO)_12_] molecules is different, just like the packing in the crystal.

### Experimental

All experiments were carried out under an atmosphere of purified, oxygen-free and dry nitrogen using standard Schlenk techniques. THF and hexane were dried and freshly distilled prior to use according to standard methods. The commercially available products paraformaldehyde, [Fe(CO)_5_], LiBEt_3_H, F_3_CCOOH and C_5_H_9_NH_2_ were of reagent grade and were used as received. The starting material [Fe_2_S_2_(CO)_6_] was prepared according to the literature.

[Fe_2_S_2_(CO)_6_] (1 mmol, 0.344 g) was dissolved in dry THF (40 ml) under a nitrogen atmosphere and then cooled to 195 K with acetone and liquid nitrogen. After the solution was stirred for 30 minutes, LiBEt_3_H (2 mmol) was added dropwise very slowly. At the midpoint of the addition, the color of the reaction mixture turned from red to dark green; for the rest of addition it remained green. After another 30 minutes, F_3_CCOOH (2 mmol, 0.149 ml) was added. The new mixture was stirred for an additional hour. The cool solution was added to a mixture of paraformaldehyde (40 mmol, 1.2 g) and C_5_H_9_NH_2_ (1 mmol, 1.98 ml) in THF which had been stirred for 10 h and cooled to 273 K. The last mixture was stirred for 24 h and the majority of the solvent was evaporated under vacuum. The remaining residual was filtered through silica gel. A red fraction was collected by elution with hexane. Recrystallization of the crude product from fresh distilled pentane in a fridge at 253 K for several days gave the title complex as a by-product in low and varing yields (<5%).

### Refinement

Hydrogen atoms were placed at idealized positions and allowed to ride on their parent atoms, with CH_2_ and CH_3_ bonds set equal to 0.97 and 0.96 Å, respectively and *U*_iso_(H)=1.2*U*_eq_(C) for hydrogen atoms of C7, and U_iso_ (H)=1.5U_eq_ (C) for hydrogen atoms of C8. The highest residual peak was located at 0.88 Å from S1.

### Crystal data

**Table:**

[Fe_4_(C_2_H_5_S)_2_(S_2_)(CO)_12_]	*Z* = 1
*M**_r_* = 745.88	*F*(000) = 370
Triclinic, *P*1	*D*_x_ = 1.826 Mg m^−^^3^
Hall symbol: -P 1	Mo *K*α radiation, λ = 0.71073 Å
*a* = 8.365 (4) Å	Cell parameters from 1200 reflections
*b* = 9.296 (5) Å	θ = 2.1–27.5°
*c* = 10.209 (5) Å	µ = 2.46 mm^−^^1^
α = 87.57 (2)°	*T* = 293 K
β = 70.082 (17)°	Prism, orange
γ = 66.103 (17)°	0.15 × 0.12 × 0.03 mm
*V* = 678.2 (6) Å^3^	

### Data collection

**Table:**

Rigaku Mercury70 CCD diffractometer	3060 independent reflections
Radiation source: fine-focus sealed tube	1939 reflections with *I* > 2σ(*I*)
graphite	*R*_int_ = 0.036
CCD_Profile_fitting scans	θ_max_ = 27.5°, θ_min_ = 2.4°
Absorption correction: multi-scan (*CrystalClear*; Rigaku, 2002)	*h* = −10→10
*T*_min_ = 0.771, *T*_max_ = 1.000	*k* = −11→11
5323 measured reflections	*l* = −12→13

### Refinement

**Table:**

Refinement on *F*^2^	Primary atom site location: structure-invariant direct methods
Least-squares matrix: full	Secondary atom site location: difference Fourier map
*R*[*F*^2^ > 2σ(*F*^2^)] = 0.048	Hydrogen site location: inferred from neighbouring sites
*wR*(*F*^2^) = 0.100	H-atom parameters constrained
*S* = 0.99	*w* = 1/[σ^2^(*F*_o_^2^) + (0.0419*P*)^2^] where *P* = (*F*_o_^2^ + 2*F*_c_^2^)/3
3060 reflections	(Δ/σ)_max_ < 0.001
163 parameters	Δρ_max_ = 0.39 e Å^−^^3^
0 restraints	Δρ_min_ = −0.42 e Å^−^^3^

### Special details

**Table:**

Geometry. All e.s.d.'s (except the e.s.d. in the dihedral angle between two l.s. planes) are estimated using the full covariance matrix. The cell e.s.d.'s are taken into account individually in the estimation of e.s.d.'s in distances, angles and torsion angles; correlations between e.s.d.'s in cell parameters are only used when they are defined by crystal symmetry. An approximate (isotropic) treatment of cell e.s.d.'s is used for estimating e.s.d.'s involving l.s. planes.
Refinement. Refinement of *F*^2^ against ALL reflections. The weighted *R*-factor *wR* and goodness of fit *S* are based on *F*^2^, conventional *R*-factors *R* are based on *F*, with *F* set to zero for negative *F*^2^. The threshold expression of *F*^2^ > σ(*F*^2^) is used only for calculating *R*-factors(gt) *etc*. and is not relevant to the choice of reflections for refinement. *R*-factors based on *F*^2^ are statistically about twice as large as those based on *F*, and *R*- factors based on ALL data will be even larger.

### Fractional atomic coordinates and isotropic or equivalent isotropic displacement parameters (Å^2^)

**Table:**

	*x*	*y*	*z*	*U*_iso_*/*U*_eq_	
Fe1	0.31028 (8)	1.00320 (7)	0.78114 (6)	0.03542 (19)	
Fe2	0.33627 (9)	0.73745 (7)	0.86174 (7)	0.03887 (19)	
S1	0.06448 (15)	0.94806 (12)	0.89480 (11)	0.0364 (3)	
S2	0.37128 (16)	0.79317 (13)	0.63756 (12)	0.0425 (3)	
O2	0.2904 (5)	1.1642 (4)	1.0304 (4)	0.0659 (10)	
O1	0.7064 (5)	0.9407 (5)	0.6498 (4)	0.0765 (12)	
O3	0.1430 (6)	1.2810 (4)	0.6441 (4)	0.0801 (13)	
O5	0.3256 (6)	0.7693 (4)	1.1491 (4)	0.0767 (12)	
C1	0.5507 (7)	0.9675 (5)	0.6996 (5)	0.0485 (12)	
O6	0.7439 (5)	0.5643 (5)	0.7670 (5)	0.0864 (14)	
C3	0.2065 (7)	1.1746 (6)	0.6958 (5)	0.0477 (12)	
C6	0.5855 (8)	0.6296 (6)	0.8033 (5)	0.0559 (14)	
C2	0.2944 (6)	1.1039 (5)	0.9341 (5)	0.0444 (11)	
C5	0.3261 (7)	0.7589 (5)	1.0381 (6)	0.0527 (13)	
C4	0.2657 (8)	0.5763 (6)	0.8735 (6)	0.0601 (14)	
O4	0.2224 (8)	0.4741 (5)	0.8805 (6)	0.1078 (17)	
C7	0.1744 (7)	0.8070 (6)	0.5873 (5)	0.0546 (13)	
H7A	0.0604	0.8465	0.6691	0.065*	
H7B	0.1589	0.8820	0.5186	0.065*	
C8	0.2037 (11)	0.6549 (7)	0.5290 (9)	0.124 (3)	
H8A	0.0984	0.6666	0.5044	0.187*	
H8B	0.2173	0.5809	0.5972	0.187*	
H8C	0.3149	0.6166	0.4468	0.187*	

### Atomic displacement parameters (Å^2^)

**Table:**

	*U*^11^	*U*^22^	*U*^33^	*U*^12^	*U*^13^	*U*^23^
Fe1	0.0322 (4)	0.0369 (4)	0.0375 (4)	−0.0163 (3)	−0.0103 (3)	0.0028 (3)
Fe2	0.0377 (4)	0.0317 (3)	0.0461 (4)	−0.0122 (3)	−0.0159 (3)	0.0021 (3)
S1	0.0297 (6)	0.0386 (6)	0.0380 (6)	−0.0142 (5)	−0.0079 (5)	−0.0008 (5)
S2	0.0354 (6)	0.0472 (7)	0.0405 (6)	−0.0142 (5)	−0.0110 (5)	−0.0050 (5)
O2	0.078 (3)	0.079 (2)	0.058 (2)	−0.048 (2)	−0.024 (2)	−0.0046 (19)
O1	0.044 (2)	0.109 (3)	0.076 (3)	−0.041 (2)	−0.008 (2)	0.001 (2)
O3	0.086 (3)	0.063 (2)	0.086 (3)	−0.018 (2)	−0.042 (3)	0.027 (2)
O5	0.092 (3)	0.077 (3)	0.057 (2)	−0.020 (2)	−0.040 (2)	0.009 (2)
C1	0.045 (3)	0.055 (3)	0.046 (3)	−0.025 (3)	−0.011 (2)	0.005 (2)
O6	0.045 (2)	0.079 (3)	0.110 (3)	0.004 (2)	−0.029 (2)	−0.025 (2)
C3	0.052 (3)	0.048 (3)	0.045 (3)	−0.022 (2)	−0.019 (2)	0.008 (2)
C6	0.049 (3)	0.049 (3)	0.066 (4)	−0.010 (3)	−0.026 (3)	−0.005 (3)
C2	0.037 (3)	0.049 (3)	0.053 (3)	−0.024 (2)	−0.015 (2)	0.007 (2)
C5	0.053 (3)	0.043 (3)	0.056 (3)	−0.010 (2)	−0.023 (3)	0.008 (2)
C4	0.075 (4)	0.047 (3)	0.071 (4)	−0.030 (3)	−0.036 (3)	0.015 (3)
O4	0.150 (5)	0.074 (3)	0.149 (4)	−0.078 (3)	−0.076 (4)	0.033 (3)
C7	0.050 (3)	0.062 (3)	0.058 (3)	−0.021 (3)	−0.027 (3)	−0.001 (3)
C8	0.131 (7)	0.086 (5)	0.194 (9)	−0.029 (5)	−0.116 (7)	−0.027 (5)

### Geometric parameters (Å, °)

**Table:**

Fe1—C1	1.786 (5)	O2—C2	1.137 (5)
Fe1—C2	1.793 (5)	O1—C1	1.145 (5)
Fe1—C3	1.824 (5)	O3—C3	1.123 (5)
Fe1—S1	2.2393 (15)	O5—C5	1.140 (6)
Fe1—S2	2.2688 (16)	O6—C6	1.138 (6)
Fe1—Fe2	2.5183 (15)	C4—O4	1.138 (6)
Fe2—C5	1.789 (5)	C7—C8	1.451 (7)
Fe2—C6	1.794 (5)	C7—H7A	0.9700
Fe2—C4	1.806 (5)	C7—H7B	0.9700
Fe2—S1	2.2457 (15)	C8—H8A	0.9600
Fe2—S2	2.2711 (18)	C8—H8B	0.9600
S1—S1^i^	2.113 (2)	C8—H8C	0.9600
S2—C7	1.842 (5)		
			
C1—Fe1—C2	90.8 (2)	C4—Fe2—Fe1	149.28 (16)
C1—Fe1—C3	99.4 (2)	S1—Fe2—Fe1	55.72 (4)
C2—Fe1—C3	99.1 (2)	S2—Fe2—Fe1	56.27 (4)
C1—Fe1—S1	157.40 (15)	S1^i^—S1—Fe1	111.12 (8)
C2—Fe1—S1	94.02 (15)	S1^i^—S1—Fe2	111.36 (8)
C3—Fe1—S1	101.64 (16)	Fe1—S1—Fe2	68.32 (5)
C1—Fe1—S2	87.52 (16)	C7—S2—Fe1	114.87 (16)
C2—Fe1—S2	156.70 (15)	C7—S2—Fe2	113.05 (18)
C3—Fe1—S2	104.06 (16)	Fe1—S2—Fe2	67.38 (5)
S1—Fe1—S2	79.39 (5)	O1—C1—Fe1	177.8 (5)
C1—Fe1—Fe2	101.44 (15)	O3—C3—Fe1	179.3 (5)
C2—Fe1—Fe2	101.42 (15)	O6—C6—Fe2	178.4 (5)
C3—Fe1—Fe2	150.37 (16)	O2—C2—Fe1	177.7 (4)
S1—Fe1—Fe2	55.96 (4)	O5—C5—Fe2	177.8 (5)
S2—Fe1—Fe2	56.35 (5)	O4—C4—Fe2	179.6 (6)
C5—Fe2—C6	90.6 (2)	C8—C7—S2	111.8 (4)
C5—Fe2—C4	99.0 (2)	C8—C7—H7A	109.3
C6—Fe2—C4	100.4 (2)	S2—C7—H7A	109.3
C5—Fe2—S1	94.44 (15)	C8—C7—H7B	109.3
C6—Fe2—S1	156.35 (18)	S2—C7—H7B	109.3
C4—Fe2—S1	101.55 (18)	H7A—C7—H7B	107.9
C5—Fe2—S2	158.11 (17)	C7—C8—H8A	109.5
C6—Fe2—S2	87.54 (17)	C7—C8—H8B	109.5
C4—Fe2—S2	102.75 (18)	H8A—C8—H8B	109.5
S1—Fe2—S2	79.21 (5)	C7—C8—H8C	109.5
C5—Fe2—Fe1	102.83 (16)	H8A—C8—H8C	109.5
C6—Fe2—Fe1	100.63 (17)	H8B—C8—H8C	109.5

Symmetry codes: (i) −*x*, −*y*+2, −*z*+2.
